# Rheumatoid Arthritis Patients, Both Newly Diagnosed and Methotrexate Treated, Show More DNA Methylation Differences in CD4^+^ Memory Than in CD4^+^ Naïve T Cells

**DOI:** 10.3389/fimmu.2020.00194

**Published:** 2020-02-14

**Authors:** Kari Guderud, Line H. Sunde, Siri T. Flåm, Marthe T. Mæhlen, Maria D. Mjaavatten, Siri Lillegraven, Anna-Birgitte Aga, Ida M. Evenrød, Ellen S. Norli, Bettina K. Andreassen, Sören Franzenburg, Andre Franke, Espen A. Haavardsholm, Simon Rayner, Kristina Gervin, Benedicte A. Lie

**Affiliations:** ^1^Department of Medical Genetics, University of Oslo and Oslo University Hospital, Oslo, Norway; ^2^K. G. Jebsen Inflammation Research Centre, University of Oslo, Oslo, Norway; ^3^Department of Rheumatology, Diakonhjemmet Hospital, Oslo, Norway; ^4^Department of Rheumatology, Martina Hansens Hospital, Bærum, Norway; ^5^Department of Research, Cancer Registry of Norway, Institute for Population-Based Research, Oslo, Norway; ^6^Institute of Clinical Molecular Biology, Christian-Albrechts-University of Kiel, Kiel, Germany; ^7^Pharmacoepidemiology and Drug Safety Research Group, Department of Pharmacy, School of Pharmacy, University of Oslo, Oslo, Norway; ^8^PharmaTox Strategic Research Initiative, Faculty of Mathematics and Natural Sciences, University of Oslo, Oslo, Norway

**Keywords:** rheumatoid arthritis, T cells, CD4 memory, CD4 naïve, DNA methylation, RRBS, epigenetic, methotrexate

## Abstract

**Background:** Differences in DNA methylation have been reported in B and T lymphocyte populations, including CD4^+^ T cells, isolated from rheumatoid arthritis (RA) patients when compared to healthy controls. CD4^+^ T cells are a heterogeneous cell type with subpopulations displaying distinct DNA methylation patterns. In this study, we investigated DNA methylation using reduced representation bisulfite sequencing in two CD4^+^ T cell populations (CD4^+^ memory and naïve cells) in three groups: newly diagnosed, disease modifying antirheumatic drugs (DMARD) naïve RA patients (*N* = 11), methotrexate (MTX) treated RA patients (*N* = 18), and healthy controls (*N* = 9) matched for age, gender and smoking status.

**Results:** Analyses of these data revealed significantly more differentially methylated positions (DMPs) in CD4^+^ memory than in CD4^+^ naïve T cells (904 vs. 19 DMPs) in RA patients compared to controls. The majority of DMPs (72%) identified in newly diagnosed and DMARD naïve RA patients with active disease showed increased DNA methylation (39 DMPs), whereas most DMPs (80%) identified in the MTX treated RA patients in remission displayed decreased DNA methylation (694 DMPs). Interestingly, we also found that about one third of the 101 known RA risk loci overlapped (±500 kb) with the DMPs. Notably, introns of the *UBASH3A* gene harbor both the lead RA risk SNP and two DMPs in CD4^+^ memory T cells.

**Conclusion:** Our results suggest that RA associated DNA methylation differences vary between the two T cell subsets, but are also influenced by RA characteristics such as disease activity, disease duration and/or MTX treatment.

## Introduction

Rheumatoid arthritis (RA) is a chronic autoimmune disease that causes pain and swelling of multiple joints in the body. The underlying disease mechanisms are believed to involve a complex interplay between common genetic and environmental factors. The heritability of RA has been estimated to be ~50% for anti-citrullinated protein antibody (ACPA) positive RA and ~20% for ACPA negative RA in a large familial aggregation study (1). Genome-wide association studies (GWAS) have identified more than 100 RA risk loci, mostly conferring risk to ACPA positive RA, marked by lead single nucleotide polymorphisms (SNPs) across various populations (2). The risk SNPs have small effect sizes, and only explain parts of heritability in RA. Environmental and epigenetic factors are also thought to be involved in the RA disease pathogenesis (3) of which smoking is the only established environmental risk factor (4, 5).

Epigenetic modifications are important for regulation and maintenance of cell type specific biological functions, and alterations in the epigenome have been found to be associated with RA (6). The most studied epigenetic modification in humans is DNA methylation of cytosine followed by a guanine at so-called CpG sites (CpGs). CpGs are often clustered in regions called CpG islands (CGIs), which frequently overlap gene promoters (7). DNA methylation in promotor regions is usually negatively correlated with transcription of the nearby gene (8).

A wide range of immune cells has been implicated in the pathogenesis of RA. One of the most widely used drugs for treatment of RA, methotrexate (MTX) (9), acts as an immunosuppressant in proliferating cells (10), and of these, the most relevant cell population for RA is CD4^+^ T cells (11). Interestingly, the RA risk loci are enriched in accessible chromatin regions (H3K4me3 peaks) in T cells, including both CD4^+^ naïve and CD4^+^ memory T cells (2). Studies have identified cell type specific DNA methylation differences in B (CD19^+^) and T (CD3^+^) lymphocytes (12, 13), as well as CD4^+^ T cells subsets (14, 15) isolated from RA patients compared to healthy controls.

However, memory and naïve CD4^+^ T cells also display distinct genome-wide and gene-specific DNA methylation patterns as a result of normal differentiation (16); hence analyses of bulk T cells may be confounded by different proportions of naïve and memory T cells. Given the recent observations that CD4^+^ T cell subset distributions are abnormal both in treatment naïve RA patients and in RA patients who has undergone MTX treatment (17) methylation profiles for distinct CD4^+^ T cell subpopulations should be investigated separately.

Methylation levels have so far only been assessed by array-based methods in RA, however reduced representation bisulfite sequencing (RRBS) using next generation sequencers allows for an interrogation of even more CpG sites. RRBS enriches for CpG dinucleotides by utilizes the restriction enzyme MspI (C^∧^CGG) to digest the DNA sample before bisulfite conversion and sequencing.

In this study, we aimed to investigate whether we could detect DNA methylation differences in primary naïve and memory CD4^+^ T cells from RA patients. To do this, we conducted an epigenome-wide association study using RRBS on isolated T cell populations from two different RA cohorts; (1) disease modifying anti-rheumatic drug (DMARD) naïve RA patients with active disease and (2) MTX-treated RA patients who had been in remission for >12 months. The two cohorts were compared to matched healthy controls.

## Materials and Methods

### Subjects

A total of 29 RA patients diagnosed according to the 2010 American College of Rheumatology/European League Against Rheumatism (ACR/EULAR) RA classification criteria (18) were included in this study. Patients were recruited from Diakonhjemmet Hospital (*N* = 28) and Martina Hansen's Hospital (*N* = 1) between 2014 and 2016 in two clinical cohorts: (1) The Norwegian Very Early Arthritis Clinic (NOR-VEAC) observational study (ISRCTN05526276) and (2) The Assessing Withdrawal of Disease-Modifying Anti-rheumatic Drugs in Rheumatoid Arthritis (ARCTIC REWIND) trial (ClinicalTrials.gov identifier NCT01881308). The newly diagnosed patients from NOR-VEAC (*N* = 11) had active disease at inclusion and blood was drawn before treatment with DMARD. To our knowledge, the patients did not receive treatment with prednisolone before blood sampling. Healthy controls recruited from the Norwegian Bone Marrow Register (*N* = 9) were matched on age (±3 years), gender, smoking status and residential area to the patients from the NOR-VEAC cohort. The MTX-treated patients from ARCTIC REWIND (*N* = 18) had symptom duration of <5 years, and the disease activity in the patients was monitored by disease activity score 28 (DAS28) (19), a composite score developed to measure disease activity in RA patients. To be included in ARCTIC REWIND, a DAS28 score at <2.6 must have been achieved for at least 12 months and stable over the last 12 months.

### Clinical Data and Statistical Comparison of the Cohorts

At the time of blood sample withdrawal, a rheumatologist clinically examined the patients. Available clinical data included DAS28 score, rheumatoid factor (RF) status, ACPA status, C-reactive protein (CRP), erythrocyte sedimentation rate (ESR), gender, age, smoking status and medication (Table 1). In addition to MTX, 6 of the 11 newly diagnosed patients and 8 of the 18 MTX treated patients used other medications regularly (see Supplementary Table 1).

**Table 1:** Demographic and clinical characteristics of the newly diagnosed DMARD naïve RA patient cohort, the MTX treated RA patients in remission cohort and the healthy controls included in the study.

	RA-DMARD naïve^*^ **(*N* = 11)**	**RA-MTX treated** **(*N* = 18)**	**Controls** **(*N* = 9)**	***p*****-values**	
**Matching criteria**				RA-DMARD naïve^*^ **vs. controls**	**RA-MTX treated vs. controls**
Female*; N* (%)	8 (72.7)	15 (83.3)	7 (77.8)	0.80	0.73
Age; mean yrs *N* [SD]	56.1 [12.2]	49.1 [13.7]	52.4 [9.1]	0.47	0.52
Smoking; *N* (%)	8 (72.7)	13 (72.2)	6 (66.7)	0.77	0.77
**Disease characteristics**				RA-DMARD naïve^*^ **vs. RA-MTX treated**	
Disease duration; mean yrs [SD]	0 [0.0]	2.6 [0.7]	N/A	<0.001	
RF positive; *N* (%)	8 (72.7)	12 (66.7)	N/A	0.73	
ACPA positive; *N* (%)	10 (90.9)	15 (83.3)	N/A	0.57	
DAS28; mean [SD]	5.2 [1.7]	1.6 [0.6]	N/A	<0.001	
CRP, mg/L; mean [SD]	21.6 [29.9]	2.2 [2.5]	N/A	0.01	
ESR, mm/h: mean [SD]	32 [17.9]	10.7 [7.7]	N/A	<0.001	
MTX dosage, mg; mean [SD]	0 [0.0]	19.4 [4.6]	N/A	<0.001	

* *To the best of our knowledge, the newly diagnosed patients did not receive prednisolone before inclusion in the study*.

*RA, Rheumatoid arthritis; DMARD, Disease modifying antirheumatic drug; MTX, Methotrexate; Yrs, Years; SD, Standard deviation; RF, Rheumatoid factor; ACPA, Anti-citrullinated peptide antibody; DAS28, Disease activity score in 28 joints; CRP, C-reactive protein; ESR, Erythrocyte sedimentation rate; N/A, Not applicable*.

To statistically compare the demographic and clinical characteristics between the patient cohorts and the healthy controls, we used the statistical program SPSS version 25 and R version 3.3.4. Independent *t*-tests were used for the continuous variables and chi-squared tests were used for the categorical variables.

### Isolation of Immune Cells and DNA Extraction

The isolation of T cells from all samples was initiated within 30 min after blood sampling. 200 ml whole blood was collected using a blood bag (Fresenius Kabi, Oslo, Norway) pre-filled with 2 ml EDTA (Thermo Fisher Scientific Inc, Massachusetts, USA) and directly diluted in 300 ml PBS (PBS without MgCl, Thermo Fisher Scientific Inc) with 1 ml EDTA and 10 ml fetal bovine serum (BioNordika, Oslo, Norway). The blood-PBS solution was transferred to 50 ml SepMate™ tubes (STEMcell Technologies, Vancouver, British Columbia, Canada) pre-filled with 14 ml Lymphoprep (Alere, Massachusetts, USA) and centrifuged following the company's recommendations. The peripheral blood mononuclear cells (PBMC) enriched cell suspension was then washed with PBS (0.4% EDTA). The CD4^+^ cells were isolated using EasySep™ Human CD4^+^CD25^+^ T Cell Isolation Kit (STEMcell Technologies) with enrichment of CD4^+^ cells, followed by depletion of CD25^+^ cells. The CD4^+^CD25^−^ cells were separated into CD4^+^ memory (CD8^−^, CD14^−^, CD16^−^, CD19^−^, CD20^−^, CD36^−^, CD56^−^, CD123^−^, TCR gamma / delta, CD66b, glycophorinA, CD25^−^, CD45RO^+^) and CD4^+^ naïve (CD8^−^, CD14^−^, CD16^−^, CD19^−^, CD20^−^, CD36^−^, CD56^−^, CD123^−^, TCR gamma / delta, CD66b, glycophorinA, CD25^−^, CD45RO^−^) cells [EasySep™ PE Selection Kit and CD45RO-PE (BioLegend, San Diego; USA)]. Isolated cells were tested for purity and viability by using BD Accuri™ C6 Cytometer (BD Biosciences, New Jersey, USA). We used Fluorescence Minus One (FMO) to set the gates.

DNA was isolated from CD4^+^ memory and CD4^+^ naïve T cells using RNA/DNA/Protein Purification Plus Kit (Norgen Biotek Corp, Ontario, Canada), and cleaned using QIAamp DNA Micro Kit (Qiagen, Hilden, Germany). The extracted DNA from the CD4^+^ naïve cells was treated with proteinase K and RNase A (Master-Pure Complete DNA & RNA Purification kit, Epicentre), and clean up was performed using 1.8x Agencourt Ampure XP beads (Thermo Fisher Scientific). The extracted DNA from CD4^+^ memory cells was treated with 10 mg/ml proteinase K (Sigma-Aldrich, Missouri, USA) and cleaned with Genomic DNA Clean & Concentrator columns (Zymo Research, California, USA). Extracted and Proteinase K-treated DNA from both cell types was quantified and qualified by Qubit 2.0 fluormeter dsDNA HS Assay Kit (Thermo Fisher Scientific Inc) and NanoDrop (Model ND1000, software v3.0.0, Thermo Fisher Scientific Inc.).

### Library Preparation for Multiplexed Reduced Representation Bisulfite Sequencing (mRRBS)

The DNA extracted from the CD4^+^ naïve cells was prepped for mRRBS on an Illumina HiSeq3000 according to the Boyle et al. mRRBS protocol (20) using the Diagenode Premium RRBS kit (Diagenode, Seraing, Belgium) (21). The mRRBS libraries (*N* = 48) were sequenced with indexes from the Diagenode Premium RRBS kit as 50 base pair, single end reads with 20% PhiX spike in and 6 samples per lane.

The CD4^+^ memory cells were prepped for mRRBS on Illumina HiSeq2500 at the Institute of Clinical Molecular Biology, Christian-Albrechts-University of Kiel, using an in-house protocol based on Boyle et al. (20) and Gu et al. (22). The libraries *(N* = 48) were sequenced as 50 base pair single end reads with 10% PhiX spike in and 6 samples per lane. Achieved Phred quality score was >28 in all retained reads from the mRRBS sequencing.

One sample (technical replicate) was sequenced on both platforms (HiSeq 2500 and HiSeq 3000) and was compared to determine how the different sequencing platforms would impact the results. The correlation was 0.9341 for this sample, and hence we found the results to be comparable (Table 2).

**Table 2:** Correlation between HiSeq machines and cell types.

	**Controls**	**RA DMARDs naïve**	**RA MTX treated**	**Replication sample on both HiSeqs**
**HiSeq 2500** Mean correlation within group	0.9195	0.9450	0.9438	-
**HiSeq 3000** Mean correlation within group	0.8940	0.8707	0.9318	-
Correlation (%)	97.45	93.37	98.80	93.41

*RA, Rheumatoid arthritis; DMARDs, Disease modifying antirheumatic drugs; MTX, Methotrexate*.

The generated methylation data has been deposited to Gene Expression Omnibus (accession number GSE135770).

### DNA Methylation Analysis

#### Sequencing Alignment and Quality Control

DNA methylation analyses were carried out using a combination of Unix, Python, Java and R (23) with Bioconductor (v2.10) (24). Fastq files were pre-trimmed and aligned to hg19 with a maximum of 2 mismatches per read length using BSMAP (25). The *methratio.py* script was used to calculate DNA methylation percentage per loci. Quality control of sequencing reads was initially performed using FastQC^1^. After alignment, the alignment efficiency and specificity of the reads were assessed using the *HsMetrics* and *RrbsSummaryMetrics* within the Picard software package (http://broadinstitute.github.io/picard). To reduce a possible source of bias in the analyses, we removed CpGs on the sex chromosomes and overlapping SNPs. RnBeads (26) was used for further QC analyses, including manual inspection of plots. PCA plots showed acceptable clustering, except two outliers that were removed from further analysis (i.e., two controls for CD4^+^ memory T cells in the comparison with MTX treated RA). A list of potentially polymorphic CpGs have been generated using the 2011051 release of the 1,000 Genomes project (27). To minimize the number of false positives, the data was filtered to only include CpGs with ≥10x coverage, a group size of ≥5 samples in both cases and controls and false discovery rate (FDR) adjusted *P*-value ≤ 0.05. Annotation was done using the R package *AnnotatR* (28) from Bioconductor.

#### Differential DNA Methylation

To identify differentially methylated positions (DMPs) between the newly diagnosed RA patients and controls and between the MTX treated RA patients and controls, we used a fitted linear regression model using the limma package (29) implemented in the RnBeads package (26) and the mean DNA methylation differences between groups. In the model, we adjusted for covariates, i.e., sex, age, smoking (never/ever), and ACPA status. The differential DNA methylation analyses were performed in naïve and memory CD4^+^ T cells, separately, to explore differences associated with either active RA disease or MTX treated RA in remission. QQ (quantile-quantile) plots with lambda-values and standard errors were generated in the R package GenABEL. The proportion of DMPs among the total number of tested sites between the two CD4^+^ cell populations was compared using chi-square tests.

Gene ontology (GO) analyses were performed based on the results from the comparison of methylation levels in CD4^+^ memory cells from MTX treated RA patients and controls, as a substantial amount of DMPs were identified using the R package topGO, where every gene is represented by one value, regardless of the number of sites representing a gene. For all genes represented by <1 significant DMP, the lowest *P*-value among all annotated DMPs were selected. To prioritize genes with more than one significant DMP annotation, we took the product of all significant DMPs into the GO analyses. Supplementary Table 2 shows the number of significant DMPs and the *P*-values used in the GO analysis.

Overlap between reported RA GWAS loci and the significant DMPs observed in our study was investigated by using a window size of maximum ±500 kb surrounding the SNPs reported by Yarwood et al. (30).

## Results

A flow chart of the study design and methodological steps is shown in Supplementary Figure 1.

### Characterization of Patient Cohorts

The demographic and clinical characteristics of the RA patients and controls included in this study are shown in Table 1. The control group (*N* = 9) did not differ from the two patient cohorts with respect to age, gender and smoking status (*P* > 0.4). When comparing the clinical parameters, the newly diagnosed DMARD naïve RA patients had significantly higher CRP, ESR, and DAS28 scores than the MTX treated patients (*P* ≤ 0.01). The mean (SD) disease duration after RA diagnosis for the MTX treated patient cohort was 2.6 years (0.7), with an MTX dosage at sample collection ranging from 10 to 25 mg per week with a mean of 19.4 mg (Supplementary Table 1). Other medications taken at the time of sample collection are also listed in Supplementary Table 1.

### Data Generation and Quality Assessment

Flow cytometry showed that the median purity of CD4^+^ memory T cells (CD4^+^CD45RO^+^) was 95% (range 79–99%), whereas for CD4^+^ naïve T cells (CD4^+^CD45RA^+^) the median purity was 86% (range 54–98%) (Supplementary Figure 2). The median purity did not deviate between the three cohorts. PCA plots based on the individual methylation profiles showed that the samples with lower cell type purity did not cluster differently than those with high cell type purity.

The bisulfite conversion rate was 99.7% across all cohorts for CD4^+^ memory cells, and ranged from 98.1–99.4% in the CD4^+^ naïve cell samples (Table 3). The numbers of aligned RRBS reads were >13.9 million for each subset of samples (divided by cohort and cell type), and the mean CpG coverage was >9.5 per sample (Table 3). The on target base count were >350 million across the samples and cohorts, and the mean 10x coverage ranged from 18.2–25.4%.

**Table 3:** Summary of RRBS performance for the different subsets of T cells and cohorts.

**Cell type**	**Cohort**	**Reads aligned**	**On target bases**	**Bisulfite conversion**	**1x coverage (%)**	**10x coverage (%)**	**Mean CpG coverage**
CD4^+^ naïve T cells	RA-patients DMARDs naïve	20,610,227	515,258,344	98.1	52.6	25.4	12.2
CD4^+^ naïve T cells	RA-patients MTX treated	21,193,235	513,852,255	99.3	52.5	25.1	11.7
CD4^+^ naïve T cells	Healthy controls	21,045,056	489,122,790	99.4	52.5	24.5	12.1
CD4^+^ memory T cells	RA-patients DMARDs naïve	13,977,649	359,190,451	99.7	45.8	18.5	9.7
CD4^+^ memory T cells	RA-patients MTX treated	18,677,449	437,185,779	99.7	44.0	19.6	12.0
CD4^+^ memory T cells	Healthy controls	16,545,688	371,925,046	99.7	45.3	18.2	10.5

*CD4^+^ naïve T cells were sequenced on HiSeq 3000, while CD4^+^ memory T cells were sequenced on HiSeq 2500 (see section materials and methods). Data have been generated using HS metrics and RRBS metrics from Picard tools. Median values across samples are given*.

*RA, Rheumatoid arthritis; DMARDS, Disease modifying antirheumatic drugs; MTX, Methotrexate*.

### Both RA Cohorts Displayed More DNA Methylation Differences in Memory T Cells Than in Naïve T Cells

**Figure 1:**
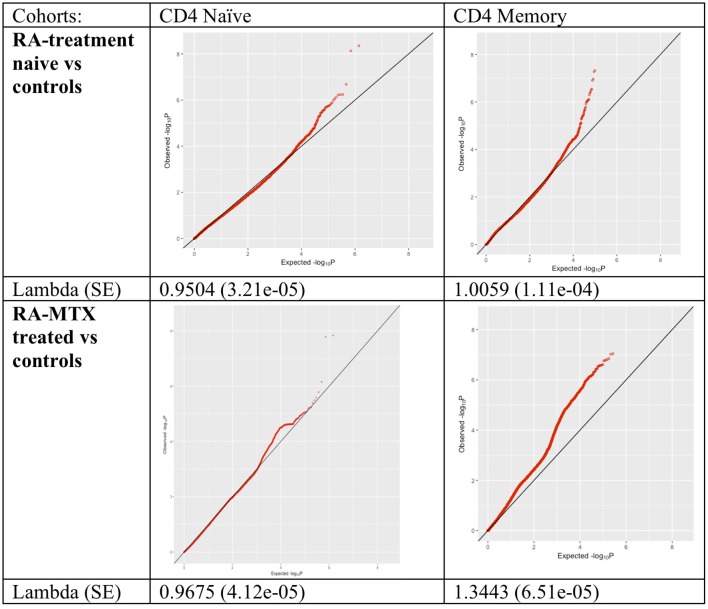
QQ-plots and lambda values of the association between RA patients and controls for all tested methylation sites in the different cohorts and cell types. RA, Rheumatoid arthritis; MTX, Methotrexate; SE, Standard error.

We systematically compared DNA methylation levels between RA patients (both newly diagnosed DMARD naïve patients and MTX treated patients) and matched healthy controls in memory and naïve CD4^+^ T cells. QQ-plots of the *P*-values for all four comparisons were distributed within the normality area (Figure 1). The global mean DNA methylation across CpGs throughout the genome was 72–73% for CD4^+^ naïve T cells and 61–62% for CD4^+^ memory T cells across all datasets (Table 4).

**Table 4:** Mean global methylation combining all CpG sites from each data set.

**Cell subset**	**Data sets**	**Controls**	**RA patients**
CD4^+^ memory T cells	MTX treated RA patients vs. controls	0.6224	0.6216
	Newly diagnosed RA patients vs. controls	0.6083	0.6052
CD4^+^ naïve T cells	MTX treated RA patients vs. controls	0.7171	0.7171
	Newly diagnosed RA patients vs. controls	0.7204	0.7279

*The sites are filtered to include CpGs with ≥10x coverage and a group size of ≥5 in both cases and controls*.

*RA, Rheumatoid arthritis; MTX, Methotrexate*.

Analyses of newly diagnosed, DMARD naïve RA patients identified 51 significant DMPs (of 933 808 tested sites) in CD4^+^ memory T cells and three significant DMPs (of 1 389 561 tested sites) in CD4^+^ naïve T cells (Table 5). Hence, significantly (*P* = 2 × 10^−9^) more of the CpG sites were differentially methylated in CD4^+^ memory T cells (0.5%) than in CD4^+^ naïve T cells (0.02%) from the newly diagnosed DMARD naïve RA patients.

**Table 5:** Filtration criteria for identification of differentially methylated CpG sites in the two RA patient cohorts compared to the healthy controls.

**Cell type**	**Cohort vs. controls**	**Number of sites**	**Mean coverage**	**Significant sites**	**Significant genes**	**Gene names**	
						**Increased methylation in RA patients**	**Decreased methylation in RA patients**
CD4^+^ naïve T cells	Newly diagnosed DMARD naïve RA patients	1 538 979	1 389 561	3	2	*SPTA1, MCC*	*-*
CD4^+^ naïve T cells	MTX treated RA patients	1 657 054	1 447 202	16	11	*SNX11, PLA1A, ZNF732, EPS15L1, STON1-GTF2A1L, STON1*,	*CACTIN, LOC100134317, RGPD3, PLEKHM1P1, SMYD3*
CD4^+^ memory T cells	Newly diagnosed DMARD naïve RA patients	984 219	933 808	51	37	*YPEL1, NELFA, CTNNA2, PARD3B, ZFP14, PHTF2, IL4I1, PGBD4, EMC7, PCSK7, RNF214, PLEKHD1, POLE2, KLHDC1, NEMF, RNF180, MIR129-2, SAMD10, PRPF6, REST, SUDS3, TRANK1, AP4E1, TEAD4, ZFP3, POM121C*	*FAM63B, MAPK8IP3, STAT5A, ZNF668, ZNF646, TMED10, CES2, GRID2IP, PLEKHA2, IQGAP3, GGCX*,
CD4^+^ memory T cells	MTX treated RA patients	1 067 343	1 035 857	853	542	*C7orf77, DENND5B, YWHAEP7, TMC5, SLCO2B1, SOX9*	*TTC37, MOXD1, TMEM129, TACC3, HIST2H3C, ISPD, LINC00862, DPP6, RORA, RNFT2, CAMKK1, TBXAS1, HIPK2, GNA15, DIAPH3, KCNJ6, LHFPL3, TRAF2, COL4A4, GALNT9, CYBA, MAP6, TBC1D3, ITPK1, PALM, TTC39B, EVI5L, MN1, S100A1, S100A13, PTPRO, FNDC3B, DLG2, UBIAD1*

*The number, names and methylation status of top 40 significant genes are also included*.

*RA, Rheumatoid arthritis; DMARD, Disease modifying antirheumatic drugs; MTX, Methotrexate; p.adj.fdr. p-value adjusted for false discovery rate*.

The MTX treated RA patients in remission showed differential DNA methylation at 853 CpGs in CD4^+^ memory T cells, and at 16 CpGs in CD4^+^ naïve T cells compared to healthy controls (Table 5). Again, there was a significant difference in the proportion of DMPs (*P* = 2x10^−68^); 853 DMPs of 1 035 857 tested sites in CD4^+^ memory T cells (8.2%) vs. 16 DMPs of 1 447 202 tested sites in CD4^+^ naïve T cells (0.11%) (Table 5).

As stated above, the MTX treated RA patients showed substantially more DMPs than the newly diagnosed DMARD naïve patients. However, it should be noted that the MTX treated RA patient cohort is larger and has more statistical power (18 vs. 11 patients). To address the effect of power between the two cohorts, we calculated how many of the DMPs in the MTX treated RA patients were significant before FDR correction in the less powerful cohort of newly diagnosed RA patients and found 92% of the DMPs in CD4^+^ memory T cells had an uncorrected *P* < 0.05. In contrast, among all tested CpGs, only 4.2% showed an uncorrected significant *P*-value. Hence, an enrichment of sites showing a trend toward association was seen among the DMPs already found in the MTX treated patients indicating that power is a likely cause of the vast differences in number of significant sites.

Nevertheless, 11 of the 20 most significant DMPs (*P* ≤ 0.0003), in any of the comparisons, were detected in newly diagnosed patients (CD4^+^ memory T cells), including the transcription factor STAT5, which is found to be an essential regulator of lymphoid development and peripheral tolerance (31) (Supplementary Table 3).

**Figure 2:**
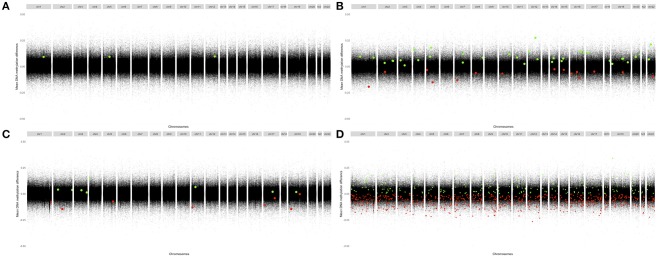
Modified Manhattan plots displaying mean change in methylation per site at their chromosomal position. Differentially methylated sites (FDR-adjusted *p* < 0.05) associated with positive changes in DNA methylation in patients relative to controls are marked in green; negative changes are marked in red. RA, Rheumatoid arthritis; MTX, Methotrexate. **(A)** CD4^+^ naïve T cells: Newly diagnosed RA patients vs. controls. **(B)** CD4^+^ memory T cells: Newly diagnosed RA patients vs. controls. **(C)** CD4^+^ naïve T cells: MTX treated RA patients vs. controls. **(D)** CD4^+^ memory T cells: MTX treated RA patients vs. controls.

The distribution of DMPs across chromosomes identified in these analyses is shown in Figure 2. None of the significant (FDR corrected) DMPs in the newly diagnosed RA patients were significant in the MTX treated RA patients. Hence, we did not, with statistical significance, identified any RA associated DMPs not influenced by disease state.

### DMPs in CD4^+^ Memory T Cells Showed Increased Methylation in DMARD Naïve Patients and Decreased Methylation in MTX Treated Patients

In newly diagnosed DMARD naïve RA patients, the identified DMPs showed increased DNA methylation, 100% of the DMPs in CD4^+^ naïve T cells and 64% of the DMPs in CD4^+^ memory T cells. In MTX treated patients in remission, 80% of the DMPs detected in CD4^+^ memory T cells displayed decreased methylation, whereas in CD4^+^ naïve T cells the proportions of DMPs associated with increased and decreased DNA methylation were equivalent. We also found that 90 of the DMPs were in close proximity (within 100 kB) to another significant DMP. Table 5 shows the top 40 genes annotated to the most significant DMPs; several annotations were possible for each DMP and the majority of the DMPs were annotated to intronic regions (Supplementary Table 3).

The significant DMPs did not overlap between either CD4^+^ T cell subset or patient cohort (Supplementary Table 3). However, when comparing genes associated and annotated to the DMPs, two genes emerged; *GRID2IP* showed decreased DNA methylation in CD4^+^ memory T cells from both RA cohorts, and *PLEKHM1P1* showed decreased DNA methylation in both memory and naïve CD4^+^ T cells from MTX treated RA patients in remission.

### DMPs Previously Associated With RA

**Figure 3:**
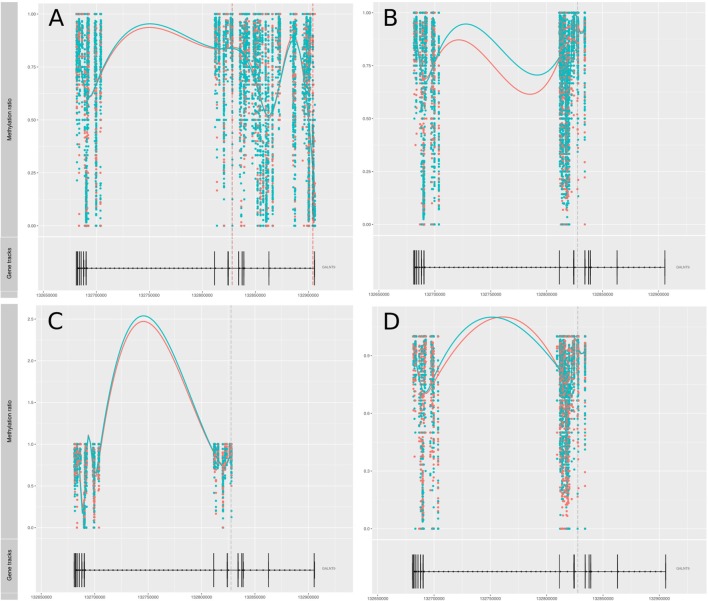
Methylation values related to the gene *GALNT9*. **(A)** RA-MTX treated patients vs. controls, CD4^+^ memory T cells. **(B)** RA-MTX treated patients vs. controls, CD4^+^ naive T cells. **(C)** Newly diagnosed RA patients vs. controls, CD4^+^ memory T cells. **(D)** Newly diagnosed RA patients vs. controls, CD4^+^ naive T cells. Teal, RA patients; Red, Controls. Gene track (bottom) showing exons and introns in *GALNT9*, with corresponding individual methylation (top) showing distribution and methylation ratio of the DMPs. Smoothed lines show an overall methylation trend between the two time-points. RA, Rheumatoid arthritis; MTX, Methotrexate; DMPs, Differentially methylated positions.

**Figure 4:**
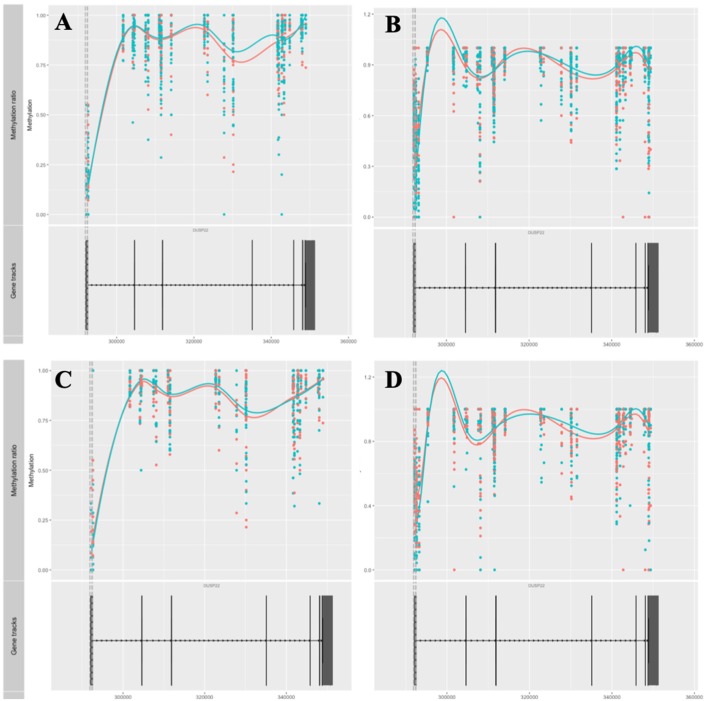
Methylation values related to the gene *DUSP22*. **(A)** RA-MTX treated patients vs. controls, CD4^+^ memory T cells. **(B)** RA-MTX treated patients vs. controls, CD4^+^ naive T cells. **(C)** Newly diagnosed RA patients vs. controls, CD4^+^ memory T cells. **(D)** Newly diagnosed RA patients vs. controls, CD4^+^ naive T cells. Teal, RA patients; Red, Controls. Gene track (bottom) showing exons and introns in *DUSP22*, with corresponding individual methylation (top) showing distribution and methylation ratio of the DMPs. Smoothed lines show an overall methylation trend between the two time-points. RA, Rheumatoid arthritis; MTX, Methotrexate; DMPs, Differentially methylated positions.

Amongst genes previously reported to have been associated with differential DNA methylation in T cells from RA patients, two genes were replicated in our study; GALNT9 and DUSP22 (12, 14, 15). *GALNT9* was significantly less methylated in our MTX treated RA patients compared to controls, but only in CD4^+^ memory T cells. The significant DMPs in our dataset were located in intron 1 which is consistent with the study by Glossop et al. (12). However, the intron 1 DMPs were only interrogated in CD4^+^ memory T cells from MTX treated RA patients. Other studies have reported various sites in intron 5 (Figure 3). The intron 5 region covering the previously reported DMPs was covered in our cohorts. Unfortunately, the other cohorts in our study showed poor coverage upstream of intron 5 and were therefore excluded from analyses. *DUSP22* was covered by CpGs across the gene in our samples, including the sites previously reported (Figure 4). However, no significant DMPs were found in this region in any of our data sets.

Of note, while the present study is based on RRBS, previous studies have analyzed DNA methylation using the Meth450K. These methods are not directly comparable and <2% of the CpGs tested in our RRBS study are present on the Meth450K (Table 6). Therefore, only 13 of the 923 significant DMPs identified in our study have been analyzed in former studies based on Meth450K data.

**Table 6:** Overlap between our tested methylation sites after RRBS and sites present on different arrays (Meth450K, EPIC, and 27K) after filtering the reads (coverage per site >10 and observed in ≥5 individuals).

		**Sites also present on Meth450 K**		**Sites also present on EPIC array**		**Sites also present on 27 K**	
	**Total sites tested in our study**	**Among our tested sites**	**Among our significant DMPs**	**Among our tested sites**	**Among our significant DMPs**	**Among our tested sites**	**Among our significant DMPs**
Newly diagnosed RA patients vs. controls (CD4^+^ naïve T cells)	1 389 561	23 659	0	27 263	0	930	0
MTX treated RA patients vs. controls (CD4^+^ naïve T cells)	1 447 202	24 823	0	28 722	0	1000	0
Newly diagnosed RA patients vs. controls (CD4^+^ memory T cells)	933 808	18 321	0	21 530	0	1004	1
MTX treated RA patients vs. controls (CD4^+^ memory T cells)	1 035 857	19 157	13	22 736	13	1047	0

*RA, Rheumatoid arthritis; RRBS, Reduced representation bisulfite sequencing; DMP, Differentially methylated position; MTX, Methotrexate*.

### RA Associated DMPs Are in Close Proximity to Genetic RA Risk Loci

We next investigated whether any of the significant DMPs overlapped with RA susceptibility loci (30) (±500 kb from the lead SNP) in CD4^+^ memory T cells (Supplementary Table 4). 32 of the 101 tested RA risk SNPs were within 500 kb of at least one DMP. Two SNPs (rs72634030-C1QBP and rs5754217-UBE2L3) were located near DMPs detected in CD4^+^ memory T cells from newly diagnosed DMARD naïve RA patients. No overlap was detected between RA SNPs and the 19 DMPs detected in CD4^+^ naïve T cells.

The CD4^+^ memory T cells from MTX treated RA patients displayed differential DNA methylation at several CpGs within the regions surrounding the RA risk SNPs. When selecting an equal number of CpGs randomly among our tested sites, we generally observed a lower number of overlapping RA SNPs, however, the association between DMPs and RA risk loci did not reach statistical significance (*P* = 0.1 after 1000 permutations). Interestingly, several of these RA risk SNPs have been annotated as eQTLs located in H3K4me3 peaks in memory T cells (i.e., *FCRL3, CD83, ANKRD55, IRF5, BLK, IKZF3, TRAF1*, and *IFNGR2)*. The distance between the lead risk SNP and the DMPs identified in our study showed substantial variation (from 4 to 434 kb). For *TRAF1*, the distance between the risk SNP and the DMP was only 4 kb, and the DMP was located within an intron of the *TRAF1* gene. This DMP was completely unmethylated in CD4^+^ memory T cells from controls, but showed detectable, but low DNA methylation levels in CD4^+^ memory T cells from MTX treated RA patients.

**Figure 5:**
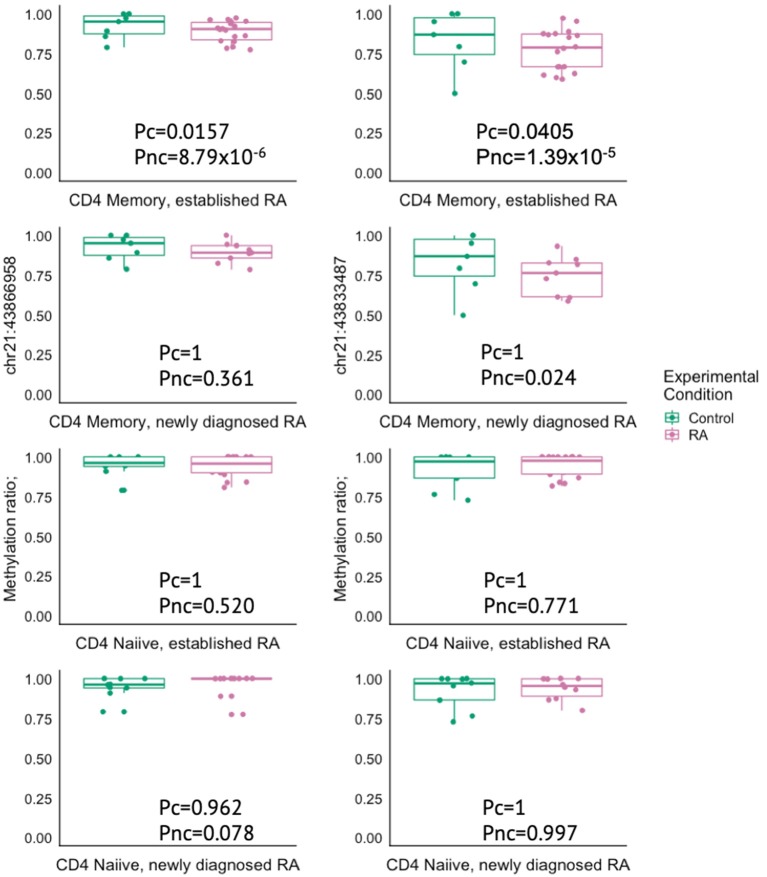
Box plots showing the methylation ratio in two different DMPs in the gene *UBASH3A* in controls (green) and in RA patients (pink) in either CD4^+^ memory or naïve T cells. RA, Rheumatoid arthritis; MTX, Methotrexate; Pc, Corrected P-value; Pnc, Not corrected *P*-value.

The gene closest to the risk SNP was often not the same as the gene closest to the DMPs within the overlapping genetic and epigenetic RA associated regions. However, both the DMP and risk SNP pointed toward the same gene for *PTPN2, IRF8, UBASH3A, TRAF1, CD83*, and *FCGR2A*. In addition, the RA risk SNP (rs1893592) is located in intron 1 of *UBASH3A*, while the two DMPs detected in our study mapped to intron 4 and 14 of *UBASH3A* (Supplementary Table 4). The *UBASH3A* DMPs were found to show reduced mean methylation in RA patients in CD4^+^ memory T cells and this difference was significant in the MTX-treated RA patients after FDR correction. The same trend was seen in the newly diagnosed DMARD naïve RA patients, but was not significant after correction for multiple testing (Figure 5).

### Gene Ontology

The CpGs tested in this study were annotated to a total of 20,449 genes, the number of CpGs per gene ranged from 1 to 1257, and the majority of the genes were covered with ≥5 CpG sites (Supplementary Table 2).

We performed GO analyses on the significant DMPs in CD4^+^ memory T cells from MTX treated patients in remission compared to controls (Table 7). The most significantly enriched GO term was skeletal system morphogenesis, which includes the terms cartilage condensation of mesenchymal cells that have been committed to differentiate into chondrocytes (GO:0001502) and bone trabecula formation (GO:0060346). Other significantly enriched GO terms included chromatin remodeling, negative regulation of response to endoplasmic reticulum stress, regulation of transcription involved in cell fate commitment, animal organ morphogenesis and blood vessel development (Table 7).

**Table 7:** Gene ontology displaying the top 10 best ranked GO terms in CD4^+^ memory T cells from MTX treated patients in remission.

**GO.ID**	**Term**	**Annotated**	**Significant**	**Classic fisher**
GO:0048705	Skeletal system morphogenesis	113	10	0.0012
GO:1903573	Negative regulation of response to endoplasmic reticulum stress	19	4	0.0017
GO:0099072	Regulation of postsynaptic membrane neurotransmitter receptor levels	32	5	0.0018
GO:0060850	Regulation of transcription involved in cell fate commitment	10	3	0.0023
GO:0009887	Animal organ morphogenesis	524	26	0.003
GO:0070534	Protein K63-linked ubiquitination	23	4	0.0036
GO:0001568	Blood vessel development	335	18	0.0061
GO:0043552	Positive regulation of phosphatidylinositol 3-kinase activity	14	3	0.0064
GO:0006338	Chromatin remodeling	79	7	0.0066
GO:0007417	Central nervous system development	450	22	0.0075

### Comparisons of Newly Diagnosed and MTX Treated RA Patients

Finally, we performed a pilot analysis, where we selected eleven of the MTX treated RA patients to best match our newly diagnosed RA patients according to age, gender and smoking status, and compared their methylation profiles in a paired test. We found 360 DMPs in CD4^+^ naïve T cells and 86 DMPs in CD4^+^ memory T cells (Supplementary Table 5). None of the genes annotated for these DMPs overlapped with genes associated with DMPs found when comparing newly diagnosed patients with controls. However, eight genes had also been detected to have DMPs when comparing MTX treated patients to controls, i.e., *SMYD3* in CD4^+^ naïve T cells and *CABLES1, B4GALNT3, EXOC6B, RASA4, MIR148A, ADCY1, MAD1L1*. The methylation of these genes is therefore likely to be caused by MTX treatment and long term RA.

## Discussion

Our epigenome-wide association study showed that CD4^+^ T cells displayed more DNA methylation differences in CD4^+^ memory T cells than in CD4^+^ naïve T cells, when comparing RA patients with controls. This was observed in both newly diagnosed DMARD naïve patients and MTX treated patients in remission, but the DMPs did not overlap between the two states of RA disease. However, due to differences in power for the two cohorts, we cannot exclude that some DMPs overlap between these two RA disease states.

Overall, we observed a higher global mean methylation in CD4^+^ naïve T cells than in CD4^+^ memory T cells, which is in accordance with observations on a whole genome-wide level, where a loss of DNA methylation occurs upon transition from naïve to memory CD4^+^ T cells (16).

Our study replicated the finding of DMPs in the gene *GALNT9*, which previously has been reported in CD4^+^ T cells (12, 14). DMPs in this gene consistently show decreased DNA methylation in RA patients across all studies, which strengthens the evidence that this gene might play a part in the pathogenesis of RA.

We did not replicate the DMPs in the other gene repeatedly reported in RA methylation studies in T and B cells, i.e., *DUSP22* (12, 13). However, it should be emphasized that the set of CpG sites investigated by our study using RRBS had very little overlap with the CpG sites interrogated by the Meth450K array used in previous studies. Even though we tested more than twice as many sites, only 20 000 of the 1 million sites measured in our study were present on the Meth450K. Such small overlap between CpGs interrogated by RRBS and Meth450K has been reported previously (32). Among our significant DMPs, only 13 were present on the Meth450K. Since we investigated a different set of CpG sites in our study, we have limited ability to compare our results to those previously published. To the best of our knowledge our study is the first to use RRBS to study methylation differences in specific T cell populations from RA patients. As such, it provides novel insight into methylation events in new genomic regions.

Previous studies in RA have generally been performed on bulk T cells or CD4^+^ T cells (12–14, 33). However, epigenomic profiling of human CD3^+^CD4^+^CD25^low^ T cell subsets from healthy individuals show distinct DNA methylation patterns *per se* between CD4^+^ naïve and memory T cells (16). One previous study, by Rhead et al. investigated DNA methylation differences in cell subpopulations from RA patients (15). In contrast to our findings, they reported more sites to be differentially methylated between RA patients and controls in CD4^+^ naïve T cells than in CD4^+^ memory T cells. This discrepancy may be caused by different methodologies such as differences in cell selection techniques (magnetic beads vs. FACS), CD25 depletion (only carried out in our study), and differences in sequencing technology including CpG positions and coverage (i.e., RRBS vs. Meth450k). In addition, differences in patient cohorts may also account for some of the differences in the results. One of the strengths of our study was the well-characterized and homogenous patient cohorts, where disease activity and treatment was known. Patients included in the study by Rhead et al., had longer disease duration, were all female and with no information regarding treatment and disease activity.

Previous studies indicate that MTX treatment reverses methylation differences in PBMC and T cells (33, 34), and that MTX treated patients have global DNA methylation levels more similar to controls than to patients with active disease. In our study, MTX treated RA patients in remission displayed more methylation differences than newly diagnosed DMARD naïve RA patients with active disease. Whether and to what extent this is a true difference is difficult to evaluate, as the sample size of our MTX treated RA patient cohort exceeded that of the newly diagnosed patients (18 vs. 11), and hence impact the statistical power. Interestingly, the *UBASH3A* gene showed the same DNA methylation levels and differences for CD4^+^ memory T cells in both MTX treated and newly diagnosed patients compared to controls, but the difference was only significant after correction in the larger cohort of MTX treated patients. Importantly, 785 (92%) of the DMPs significant after FDR correction of *P*-values in MTX treated patients had a significant *P*-value (*P* < 0.05) before correction in the newly diagnosed patients. This supports the notion that differences in statistical power may in part explain the differences between the RA cohorts.

We found that most of the differentially methylated regions were located outside promoters, in intronic regions. The biological function of intragenic DNA methylation is less clear, but it may regulate usage of alternative promoters and alternative splicing (35). In gene bodies, unlike promoters, there is not always a negative correlation between DNA methylation and transcription, in some tissues, active genes are more methylated than repressed genes. Overall, intragenic CpGs appear to be functionally important and play a role in fine tuning of gene regulation.

One third of our DMPs were located in the vicinity of genetic risk loci for RA (±500 kb away from lead SNP). Only 6 of the 38 genes overlapping or being closest to our DMPs, were consistent with genes reported from GWAS hits. However, few GWAS regions in autoimmune diseases have yet converged on a causal gene. An exception is *UBASH3A*, encoding ubiquitin-associated and SH3 domain containing protein A, which functions as a negative regulator of NF-kB signaling in T cells on stimulation of the antigen T cell receptor (36). Non-coding susceptibility alleles within *UBASH3A* are associated with several autoimmune diseases, including type 1 diabetes and RA, and are also associated with increased transcription, thereby leading to reduced transcription of interleukin-2 (IL2) in effector T cells (36). An association study by Liu et al. identified one SNP (rs1893592) in *UBASH3A* that was significantly related to DAS28, CRP level and bone erosion in RA patients (37). In line with this, we found decreased DNA methylation of two DMPs within *UBASH3A* in CD4^+^ memory T cells in MTX treated RA patients compared to controls, presumably leading to increased expression of *UBASH3A*. Interestingly, the autoimmune risk variant at rs1893592 in *UBASH3A* is associated with decreased lymphocyte percentage in the UK biobank cohort (38). Future methQTL analysis in a larger dataset, may address whether risk genotypes at the RA associated SNPs are correlated with methylation levels at these loci.

The most important limitation of our study is the small number of patients, which limits the statistical power. To compensate for this, we focused on minimizing the noise by using homogenous datasets (i.e., using two homogenous RA populations and investigating two distinct CD4^+^ T cell subsets). Unfortunately, two different laboratory procedures and sequencing instruments were used, one for each cell type. However, the sample that was included on both platforms showed 93.4% correlation.

## Conclusion

In conclusion, by focusing on specific T cell populations highly relevant for RA (CD4^+^ naïve and memory T cells) from two phenotypically distinct and homogeneous RA cohorts using an epigenome wide RRBS sequencing approach, we discovered intrinsic DNA methylation differences, including confirmation of decreased DNA methylation in CpGs within *GALNT9* in RA patients. One third of the known RA risk SNPs were located in the vicinity of our DMPs, and one of the most robust findings was for *UBASH3A*. Nevertheless, larger studies of homogenous RA patients and controls comprising bisulfite sequencing of relevant immune cell subsets are necessary to further establish the changes in DNA methylation that play a part in RA pathogenesis, drug response and disease development before the putative role of epigenetic factors can be assessed in a clinical setting.

## Data Availability Statement

The datasets supporting the conclusions of this article are included within the article and its additional files. Additional raw files and bioinformatics are available in the GEO repository, under accession number GSE135770.

## Ethics Statement

The studies involving human participants were reviewed and approved by The National Health Authorities and Regional Ethics Committee (REK# 2015/1546). The patients/participants provided their written informed consent to participate in this study.

## Author Contributions

KGu has contributed during all stages of the project, development and execution of the laboratory procedures, analyzed the data, developed the bioinformatic pipeline, result interpretation, and writing. LS has contributed during laboratory procedures, result interpretation and writing of the first draft. STF has contributed to development and execution of the laboratory procedures. MTM, MDM, SL, AB-A, EN, and EH have contributed in the recruitment and clinical examination of the patients. IE, SF, and AF have contributed to the library preparation. BA has contributed to the study design. SR and KGe have contributed to the bioinformatic pipeline and data analyses. BL has contributed to the study supervision, study design, results interpretation, and writing. All authors have read, commented, and approved the final manuscript.

### Conflict of Interest

The authors declare that the research was conducted in the absence of any commercial or financial relationships that could be construed as a potential conflict of interest.

## Footnotes

^1^FASTQC A Quality Control Tool for High Throughput Sequence Data (2015). https://www.bioinformatics.babraham.ac.uk/projects/fastqc/

## Abbreviations

ACPA: Anti-citrullinated protein antibody
ACR: American College of Rheumatology
ARCTIC REWIND: The Assessing Withdrawal of Disease-Modifying Anti-rheumatic Drugs in Rheumatoid Arthritis
CGIs: CpG islands
CpGs: CpG sites
CRP: C-reactive protein
DAS28: Disease activity score 28
DMARD: Disease modifying antirheumatic drugs
DMPs: Differentially methylated positions
ESR: Erythrocyte sedimentation rate
EULAR: European League Against Rheumatism
FDR: False discovery rate
GO: Gene ontology
GWAS: Genome-wide association studies
mRRBS: Multiplexed reduced representation bisulfite sequencing
MTX: Methotrexatel
NOR-VEAC: The Norwegian Very Early Arthritis Clinic
RA: Rheumatoid arthritis
RF: Rheumatoid factor
SD: Standard deviation
SNPs: Single nucleotide polymorphisms.
