# Supplementary material for: Rheumatoid Arthritis Patients, Both Newly Diagnosed and Methotrexate Treated, Show More DNA Methylation Differences in CD4+ Memory Than in CD4+ Naïve T Cells
Source: Front Immunol. 2020 Feb 14;11:194. doi: 10.3389/fimmu.2020.00194 (PMC7033478; doi:10.3389/fimmu.2020.00194)
Supplement: Supplementary file 1 [file Table_1.docx]

| **Table 1_v1:** Other medication reported to be taken at the time of sample collection | | | | | |  |
| --- | --- | --- | --- | --- | --- | --- |
|  |  |  |  |  |  |  |
| \| **Cohort** \| **MTX dosage** \| **Other medication** \| \| --- \| --- \| --- \| \| **Newly diagnosed DMARDs naïve RA patients** \| \| \| \| 1 \| 0 \|  \| \| 2 \| 0 \| Cetirizine, Ibuprofen \| \| 3 \| 0 \| Naproxen, Ibuprofen \| \| 4 \| 0 \| Naproxen, oral contraceptive pills \| \| 5 \| 0 \|  \| \| 6 \| 0 \| Diclofenac, Ibuprofen \| \| 7 \| 0 \| Esomeprazole, Ibuprofen, Paroxetine, Simvastatin, Tapentadol \| \| 8 \| 0 \|  \| \| 9 \| 0 \|  \| \| 10 \| 0 \| Diclofenac, Losartan, Paracetamol \| \| 11 \| 0 \|  \| \| **MTX treated RA patients in remission** \| \| \| \| 1 \| 15 \| Pizotifen \| \| 2 \| 20 \| Atorvastatin, Hydrochlorothiazide, Losartan \| \| 3 \| 15 \|  \| \| 4 \| 10 \|  \| \| 5 \| 20 \| Esomeprazole \| \| 6 \| 10 \|  \| \| 7 \| 20 \|  \| \| 8 \| 20 \|  \| \| 9 \| 25 \|  \| \| 10 \| 25 \|  \| \| 11 \|  \| Atorvastatin, Estradiol, Furosemide \| \| 12 \| 25 \|  \| \| 13 \| 20 \| Diclofenac, Levaxin \| \| 14 \| 20 \|  \| \| 15 \| 20 \| Intrauterine device, Naproxen \| \| 16 \| 20 \| Amlodipine, Losartan \| \| 17 \| 25 \| Diclofenac, Intrauterine device, Loratadine, Sumatriptan \| \| 18 \| 20 \|  \| \| **Healthy Controls** \|  \|  \| \| 1 \| 0 \|  \| \| 2 \| 0 \|  \| \| 3 \| 0 \| Candesartan \| \| 4 \| 0 \|  \| \| 5 \| 0 \|  \| \| 6 \| 0 \|  \| \| 7 \| 0 \|  \| \| 8 \| 0 \|  \| \| 9 \| 0 \|  \| | | |  |  |  | 6 |

*RA* Rheumatoid arthritis, *DMARDs* Disease modifying antirheumatic drugs, *MTX* Methotrexate
