# Supplementary material for: Rheumatoid Arthritis Patients, Both Newly Diagnosed and Methotrexate Treated, Show More DNA Methylation Differences in CD4+ Memory Than in CD4+ Naïve T Cells
Source: Front Immunol. 2020 Feb 14;11:194. doi: 10.3389/fimmu.2020.00194 (PMC7033478; doi:10.3389/fimmu.2020.00194)
Supplement: Supplementary file 6 [file Data_Sheet_1.pdf]

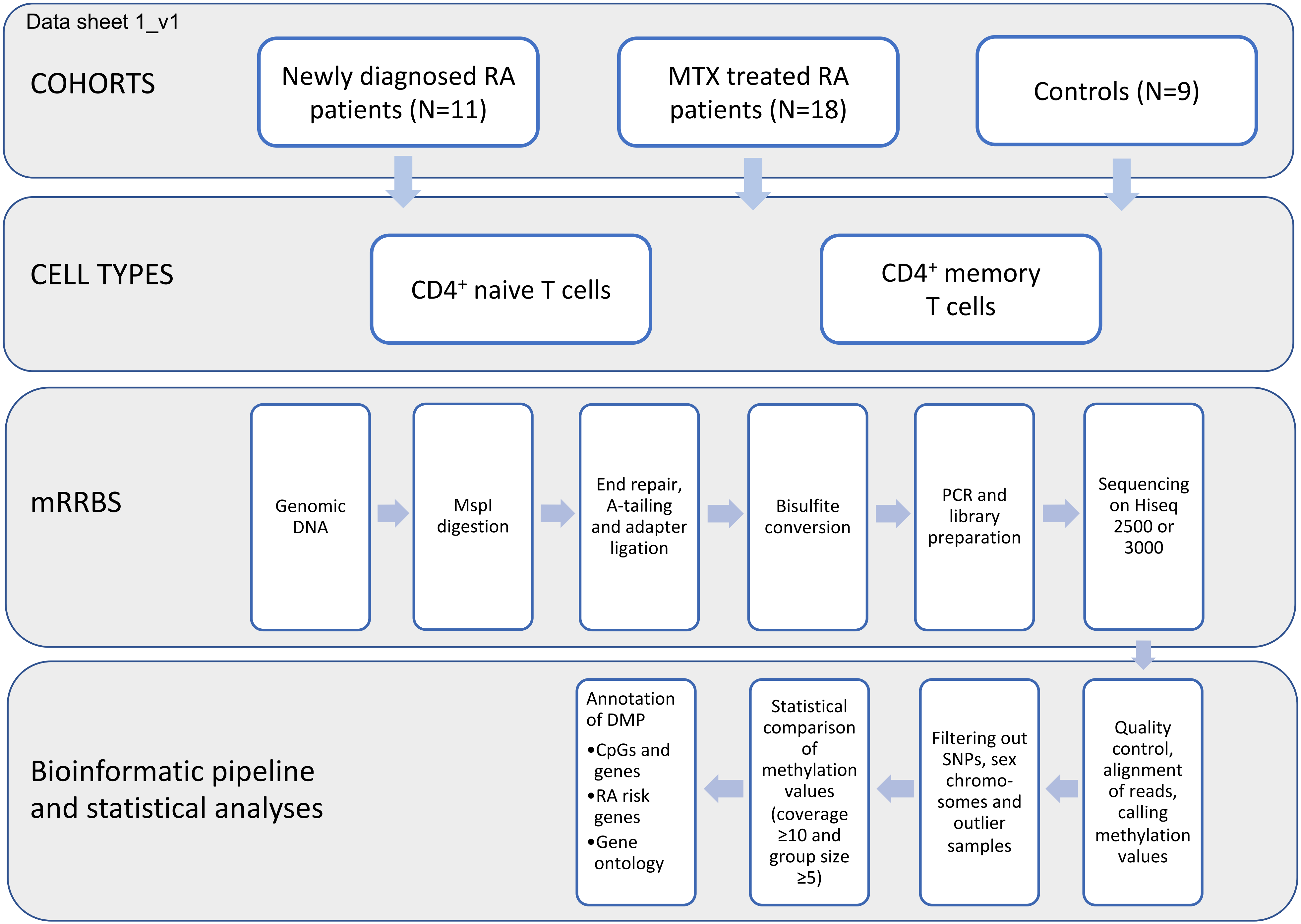
Data sheet 1_v1
COHORTS
Newly diagnosed RA patients (N=11)
MTX treated RA patients (N=18)
Controls (N=9)
CELL TYPES
CD4+ naive T cells
CD4+ memory T cells
mRRBS
Genomic DNA
MspI digestion
End repair, A-tailing and adapter ligation
Bisulfite conversion
PCR and library preparation
Sequencing on Hiseq 2500 or 3000
Bioinformatic pipeline and statistical analyses
Quality control, alignment of reads, calling methylation values
Filtering out SNPs, sex chromo-somes and outlier samples
Statistical comparison of methylation values (coverage ≥10 and group size ≥5)
Annotation of DMP
•CpGs and genes
•RA risk genes
•Gene ontology
