# Supplementary material for: Rheumatoid Arthritis Patients, Both Newly Diagnosed and Methotrexate Treated, Show More DNA Methylation Differences in CD4+ Memory Than in CD4+ Naïve T Cells
Source: Front Immunol. 2020 Feb 14;11:194. doi: 10.3389/fimmu.2020.00194 (PMC7033478; doi:10.3389/fimmu.2020.00194)
Supplement: Supplementary file 7 [file Data_Sheet_2.docx]

**Data sheet 2_v1**

**A**


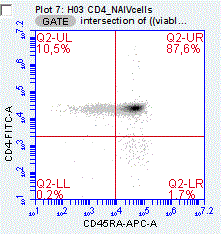


**B**


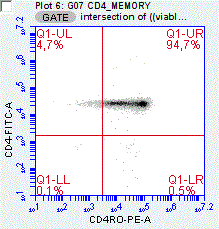


**Data sheeet 2:** Purity plots displaying sorted cells: A. CD4^+^ naive T cells. B. CD4^+^ memory T cells.
